# Editorial and Miscellaneous

**Published:** 1863-01

**Authors:** 


### EDITORIAL AND MISCELLANEOUS.

*Rush Medical College.*—The exercises of the Twentieth Annual Commencement of Rush Medical College occurred on Wednesday evening, the 21st inst. Fifty-eight gentlemen received the degree of Doctor of Medicine. Prof. Brainard conferred the diplomas, accompanying this duty with a few brief and practical observations. The usual Valedictory Address was given by Prof. R. L. Rea. Of this, we need say nothing here, as we hope in the next Number to spread it before our readers. Suffice it to say, that it worthily sustains the enviable reputation which Prof. Rea has achieved as a teacher in his particular department, and is luminous with the high professional principles which he illustrates in the rounds of private practice. Rev. Robert Collyer acted as Chaplain.

One hundred and seventy-five students have been in attendance during the course just terminated, and as the classes have been steadily increasing for several years past, it may not be unwarrantable to claim for the college very flattering prospects for the future. To sustaining and enhancing the high character and popularity it has already achieved, the Faculty will continue to lend their best energies; and they are determined, that hereafter, as heretofore, its diploma shall everywhere be received as evidence of real merit on the part of the possessor. The last graduating class was one of which any institution might well be proud.

The following list embraces the several names and theses:

Edward E. Lynn, Pathology of Otitis..............Illinois
S. L. Marston, Puerperal Convulsions............Wisconsin
F. C. Mehler, Wounds of Thorax..................Illinois
T. J. Montgomery, Evidences of Gestation.........Indiana

James Muncey, Ovarian Dropsy.....................Iowa
L. P. Y. McCoy, Functional Derangements of Uterus..Illinois
Geo. C. McFarland, Causes and Treatment of Un-
united Fractures............................Illinois
John McLean, Gunshot Wounds..................Illinois
J. W. McNeel, Signs of Pregnancy.............Wisconsin
G. Copp Noyes, Valvular Disease of the Heart....Wisconsin
C. O'Brien, Colo Rectitis.......................Wisconsin
J. W. Ogle, Bone and its Uses.................Wisconsin
Wesley Phillips, Remittent Fever..................Illinois
W. G. Pratt, Reflex Action......................Illinois
B. G. Pierce, Tendency to Death..................Illinois
J. M. Rankin, Milk Sickness......................Illinois
J. S. Ransom, Opium.............................Iowa
F. C. Robinson, Constitutional Syphilis.............Illinois
L. N. Rogers, Common Sense the Pract. Med........Illinois
J. W. Saucerman, Neuralgia..................Wisconsin
William Scott, Alcohol.........................Indiana
N. W. Sigworth, The Kidneys....................Indiana
A. L. Small, Diagnosis..........................Illinois
Lewis H. Skaggs, Dysentery......................Illinois
W. H. Smith, Mannuary Abscess................Missouri
Pembroke R. Thombs, Diphtheria...................Iowa
Gordon Andrews, Cathartics and Astringents....Wisconsin
Chas. F. Barnett, Diphtheria,.....................Indiana
E. Bishop, Dysentery and Diphtheria............Wisconsin
Ela L. Bliss, Digestia Depravate...............Wisconsin
Fred. W. Byers, Ipecacuanha...............Pennsylvania
Philo W. Chase, Sulphate Quinine...................Ohio
Jas. Cunningham, Secretion of Bile, &c...........Illinois
John W. Dean, Diphtheritis......................Indiana
Chas. F. Dilly, Rheumatic Carditis.................Iowa
Wm. B. Dunkle, Rubeola..........................Iowa
Chas. S. Elder, Physiology of Menstruation........Illinois
Francis A. Emmons, Cuculatu of Blood...........Illinois
Uriah B. Ferris, Surgery.........................Illinois
Stephen N. Fish, Epilepsy........................Illinois
Wm. M. Gregory, Diphtheria......................Illinois
Harrison H. Guthrie, Malaria.....................Illinois
Geo. F. Heideman, Acute Meningitis.............Illinois
Prier J. Herman, Puerperal Fever................Illinois
Myron Hopkins, Typhoid Fever...................Illinois
Sam'l. G. Irwin, Typhoid Fever..................Indiana
Dan'l. C. Jones, Bilious Remittent Fever...........Illinois

James Kelly, Bilious Fever........................Iowa
Chas. B. Kendall, Typhoid Fever..................Illinois
Hiram M. Keyser, Milk Sickness..................Illinois
G. Allen Lamb, Pneumonia.....................Wisconsin
Charles F. Little, Amenorrhœa....................Illinois
W. H. Thompkins, Typhoid Fever.................Illinois
J. A. Williams, Pneumonia.......................Illinois
John L. Williams, Epidemic Cholera............Wisconsin
W. T. Wilson, Typhoid Fever....................Indiana
John Zahn, Venesection in Pneumonia............Illinois

---

*To Subscribers.*—The prompt returns received in reply to the note from Prof. Ingals elicit our sincerest thanks. For those still in arrears we reserve compliment a little longer, trusting they will also soon merit it.

It will be noticed that the number of pages in the present number is somewhat reduced. We are impelled to this course in common with the press generally. Paper and labor have so largely appreciated that we must either do this or raise the price of the JOURNAL. We pledge ourselves to enlarge again just as soon as prices will warrant, be it sooner or later. Our subscribers will see at a glance that as gold has gone up, and is still rising, prices of all sorts must rise. Prices are more than one-third higher and still ascending, but we abate but one-fourth and there shall stop. All deficiencies we shall meet with our private resources.

The ensuing year we shall aim, as heretofore, to make the JOURNAL an exponent of true Medical Science and legiti mate progress. The present number has been prepared for the press under peculiar disadvantages, particularly the ill health of the acting Editor, which has prevented early issue and that attention which he will hereafter give. In lieu of long selections, it is proposed to give abstracts of all valuable articles found in our exchanges, with such comments as may seem proper. We shall aim thus to hold the mirror up to the profession. It is expected that serial articles, upon important topics, will be given both by correspondents and the Editors.

It may be observed for convenience that hereafter, as during the last two years, all editorial articles by Dr. Brainard will be subscribed by his initial. For all others, Dr. Allen alone is responsible.

Contributions to our pages are solicited from all of our subscribers who can find time (and who can not?) to use the pen. We number upon our subscription list many of the most capable minds in the North West, and we trust that these will not "hide their light under a bushel," but let it shine along the pages of the JOURNAL, so as to be seen and read by all men. *Verb. Sap.!*

---

*The Hopps' Trial.*—We have full notes of the testimony, arguments of counsel, and the Judge's charge in this very interesting case, which we shall condense for a subsequent number of the JOURNAL.

The verdict of the Jury, consigning an insane man to ignominious death, is a most startling commentary on the boasted enlightenment of the age.

With all respect to the legal profession, we must be permitted to remark, that we have never seen it so recklessly disgraced as it was by the official prosecutor in his conduct of this trial. To say that that conduct was bloodthirsty and brutal, is tame. Both the manner and the matter of his discourse were permeated in every part by the rank fumes which reach their perfection in the lowest gambling hells, the stews, and whiskey dives of the city.

In one respect, it was admirable—having not the slightest idea of the real matter involved, the brazen effrontery with which the Prosecutor launched upon a Stygian sea of low flung jokes, coarse expletives, pettifogging tricks, shyster manœuvres and filthy inuendoes—was such that truly—
"None but himself could be his parallel."

The trial, so far as the prosecution was concerned, speedily lost the character of a dignified investigation as to the guilt or innocence, responsibility or irresponsibility of the prisoner—

it became the arena where high toned gentlemen were obliged to see their cause lost, because they could not descend into the atmosphere contaminated by the pestiferous aroma exhaled from their professional antagonist.

It is understood the Jury opened their deliberations by prayer—so it has been heralded in the papers. It was a highly religious jury, and, as the tendency of extremes is to meet, so they gathered themselves into the "goodlie companie" of the Prosecuting Attorney, and awoke from their religious beatitudes to find themselves "at one" with him. With all reverence, we must say, that we had rather have heard of them as canvassing the testimony in the case—testimony which proved as clearly as the sunlight that William Hopps was an insane man, and as such wholly irresponsible for the alleged crime.

We believe in capital punishment for murder, but we put it on record, even before decision of the motion, now pending, for a new trial, that William Hopps will not be hung. You cannot hang an insane man in this past noon of the nineteenth century.

---

*The Institutes of Medicine.*—By MARTYN PAINE, A. M., M. D., LL. D., Professor of the Institutes of Medicine and Materia Medica in the University of the City of New York, &c., &c., &c. Seventh Edition. Harper & Brothers, New York, 1862.

This book is the concentrated essence and life-blood of Dr. Paine's scientific existence, and, as every student of the Metropolitan College, with which he is connected, is expected to buy one, of course, its editions are soon exhausted. Besides this, it is very cheap for the size, and thus makes a formidable figure on a library shelf at a very trivial expense. One of our Chicago ladies ordered her upholsterer to buy her a library "to match the other furniture," and several copies of the "Institutes" were shrewdly put on an upper shelf in filling the order. The man who read the "Monikins" has also secured a copy; and "the other man," who is trying for the secret of perpetual motion, thinks he has the clue now, whilst chasing Prof. P.'s ideas from references to references, among his multitudinous paragraphs. For everybody knows that this model author proves everything by his references, nothing by demonstration or detailed explanation.

The volume is capital for museum purposes, it saves so much trouble in collecting antiquities and fossils elsewhere in divers directions.

To the earnest student we need only say that this volume is utterly worthless. Its so-called principles are but the shadows from the dark ages of medicine projected over upon our times. It is a heavy drag-net filled with the ooze and slime of drowned antiquity.

The author is one striving to "ray out darkness" rather than light.

He is infallibly unjust toward every author whom he quotes or notices, whether to censure or drag to the support of his own limping, half starved dogmas.

Common literary fairness is unknown to him, and to this day, though convicted, again and again, of the grossest misrepresentations and calumnies, he never retracts. What he once asserts he stereotypes and repeats, ever after unchanged.

The Institutes of Medicine, as he would teach them, are a continuous "crime against nature." His teachings have done more to render MEDICINE a byword and a reproach than all the studied attacks upon it, or than any half score epidemics baffling treatment, since he was born.

---

*The Hospital Steward's Manual.*—For the instruction of Hospital Stewards, Ward Masters, and Attendants in their several duties. Prepared in strict accordance with existing regulations and the customs of service in the armies of the United States of America, and rendered authoritative by order of the Surgeon General. By JOSEPH JANVIER WOODWARD, M. D., Ass't. Surg. U. S. A., Member of the Acad. Nat. Sci. of

Philadelphia, &c. J. B. Lippincott & Co , Philadelphia, 1862.

This is one of the minor treatises begotten by the necessities of the time. The author and publishers have respectively performed their duties well, and produced a book every way suitable for the object announced in the title page. It should be in the hands of every Hospital Steward and Attendant.

---

*Anatomy of the Arteries of the Human Body, Descriptive and Surgical, with Descriptive Anatomy of the Heart.*—By JOHN HATCH POWER, M. D., F. R. C. S., &c. Authorized and adopted by the Surgeon General of the U. S. Army, for use in Field and General Hospitals. Philadelphia: J. B. Lippincott & Co., 1862.

The very superior dissections by the author and others are fully illustrated in this work, and we endorse the remark of the publishers, that it will be found an invaluable *vade mecum* for those engaged in the delicate operations incident to military surgeon.

---

*Visiting List.*—Lindsay & Blakiston have issued their now well known Visiting List for 1863.

---

*Consumption in New England, or Locality one of its Chief Causes.*—An Address delivered before the Massachusetts Medical Society. By HENRY J. BOWDITCH, M. D. Boston, 1862.

We alluded to this essay some time since, on the announcement of its doctrines in the Boston *Medical Journal*, soon after its delivery in May last. It is now brought out by Messrs. Ticknor & Fields, in a bulky but neat pamphlet, illustrated by maps and diagrams, with a few prefatory remarks, and an Appendix which gives the names of the sources of his information, with other valuable addenda.

Dr. Bowditch observes, that these investigations have occupied him for many years, especially the last eight. In 1855 and 1856 he enunciated these principles "tremblingly," but since then they have hardened into "stern belief." The line of thought will be better elucidated by the following extracts:

The two following propositions contain the essential points of this address:—

*First.* A residence on or near a damp soil, whether that dampness be inherent in the soil itself, or caused by percolation from adjacent ponds, rivers, meadows, marshes or springy soils, is one of the primal causes of consumption in Massachusetts, probably in New England, and possibly in other portions of the globe.

*Second.* Consumption can be checked in its career, and possibly, nay probably, prevented in some instances, by attention to this law.

I lay down now before you, as among my *Medical Axioms*, the following statements:—

1st. *Consumption is not, as some writers have contended, endemic equally in every part of New England;* but there are some localities where it is very rife, and others where it is vastly less destructive than in the State at large.

2d. *There is a law,* hitherto scarcely noticed, or but vaguely hinted at by one or two individual writers, but (as I believe) *never proved* until now, *which is one of the main causes, if not the sole cause, of this unequal topographical distribution of consumption in New England.*

3d. *This law is intimately connected with, and apparently dependent on, the humidity of the soils, on or near which stand the towns, villages, or even single houses, where consumption prevails.*

4th. *The existence of this law of soil-moisture, as one of the prime causes of consumption in New England,* can be proved, as I think, by several lines of argument, resting on actual facts obtained either from public or private records, statistical data, or the opinions of physicians, practising medicine in various parts of New England.

These lines of proof, or of argument, are drawn from the following sources:—

I. Massachusetts State Registration Reports.

II. Medical Opinion of Massachusetts, as embodied in the returns made to me, as a Committee of this Society—these returns consisting of written reports from resident physicians of one hundred and eighty-three towns.

III. Actual Statistics of deaths by consumption, received from such correspondents. Some of these statistics are but *incidentally* mentioned, while others are from towns, districted and carefully examined with reference to the relative prevalence of consumption in the different districts. In some of the most important of these, the examination was made without my correspondent or myself being aware of the existence of any law such as that which I shall present at this time.

IV. Peculiarities of certain towns and of villages in the same townships, in some of which consumption is quite prevalent, and in others much less so; these differences being connected most closely with corresponding differences in the amount of moisture of the soil of said places.

V. Certain well known houses, which, in various towns, are known by the inhabitants and physicians to have been long noted as the abode of consumption, and in some of which several families have been, during the past fifty years, cut off by the disease, without the least suspicion, on the part of the occupants, of the fatal position in which the houses were placed.

VI. Confirmatory facts, statistics and opinions from Rhode Island, Maine and New Hampshire.

VII. The medical statistics given in the Report on the health of the United States Army, strongly supporting the idea of the existence of the same law, and the operation of it over the whole of the United States.

VIII. Results of my own practice since I first became convinced of the truth of the law—said results consisting of (*a*) Statistics from my private medical records; (*b*) Results actually derived from my choice of localities for consumptive patients, based on a belief in the law.

IX. Apparent exceptions to the law.

On the basis of these results, he calls for better attention to the localization of houses, villages and cities; to the subject of under-drainage; the establishment of State Boards of Health; and in individual cases, he says, "*it will be our first duty to urge the patient to leave the spot.*"

The thanks of the Profession are due to Dr. B. for his laborious investigations, and the clearness and force with which he has presented his views—and views they are of very high importance. The hygienic inferences are obvious, and it must occur to every well-informed mind on this subject, that in appropriate hygiene and correct prophylaxis, and not in any mere drug or medicine, is to be sought the closing up of this terrible outlet of human life. We shall take occasion in an early issue to comment more at large on this important subject. Meanwhile we commend these thoughts to the earnest attention of all our readers.

---

*The People's Dental Journal:* Edited by W. W. Allport, D. D. S., and E. T. Creighton. Quarterly; 50 cts. a year in advance. Published by the Editors, No. 32 Washington St., Chicago.

The dental profession have already quite a number of journals devoted to their specialty, several of which are of very high order, but all of them intended for almost exclusively professional reading. The journal before us is especially devoted to a dissemination of knowledge of the more directly practical kind among the people. Not to make people dentists, but that they may know how to take care of their own teeth, and particularly the teeth of their children. The project is an excellent one, and if carried out in the manner developed in the present number cannot fail to be of public utility. The high professional reputation of Dr. Allport is a full guarantee that his journal will be filled with matter of sterling practical value.

We have marked an article by Dr. A. on "Causes of the decay of the Teeth," for reproduction in a subsequent issue. We wish the Editors entire success in their somewhat novel enterprise.

---

*Wisconsin Institute for the Blind.—Thirteenth Annual Report, Oct. 1st,* 1862.

This institution appears to be fulfilling its bennvolent purposes in the most satisfactory manner. About fifty pupils were in attendance during the last year. The Superintendent, Mr. Thos. H. Little, has discharged the duties of his responsible position in the most creditable and generally acceptable way. The Institute is located at Janesville.

Of the fifty cases, 14 were congenital; 4 from amaurosis; 4 from scrofula; 3 from small-pox; 2 from measles; 2 from scarlet fever; 13 from inflammation; 6 from accident; 1 from cataract, and 1 from hydrocephalus.

---

We would call attention to the advertisement of Messrs. Bliss & Sharp, Druggists, who have recently purchased from Messrs. J. H. Reed & Co. their Store, 144 Lake Street, (well known as their Retail Store.) They have made many alterations and improvements, filling up with a complete stock of Drugs, Chemicals, Instruments, and are prepared to pay particular attention to Physician's orders.

---

*Eighth Biennial Report of the Illinois State Hospital for the Insane, at Jacksonville. Dec.* 1862.

There were 302 patients remaining in the Hospital Dec. 1st, 1862. The extreme limit of accommodation as to numbers has been reached, although the wants of the State are not yet fully met.

We recommend careful perusal of the report of the enlightened and sagacious Superintendent, Dr. A. McFarland, to all those who have professional or personal relations to the unfortunate insane. We extract:

The general rule may safely be laid down, that all cases of insanity of an acute character—meaning those whose invasion is somewhat sudden—where the case is not a direct attendant on some febrile disease, are always best managed, and insured a favorable result, in an institution like this, than under the most favorable circumstances elsewhere. Yet, even here some discrimination is necessary. Where there is the least question as to the physical ability of the patient to bear removal to the institution, the advice of some competent medical man should be taken just on the eve of the journey. If this was more generally done, some of those deaths which annually occur here within ten days of arrival, would, possibly, not occur.

But with the larger proportion of cases, where the invasion of the disease is more gradual, the rule above laid down should not be so general. In a former report (Dec. 1856,) it is stated, that, "it by no means follows that the existence of insanity in certain forms implies the necessity or propriety of the removal of the individual so affected from the place of his ordinary residence. An insane hospital bears the same relation to the deranged mind that the splint and bandage do to the fractured limb. It protects it from dangerous extraneous influences, and holds it in a position to admit of the requisite medication. As there are some fractures where neither of these demands exist, so there are many cases of insanity where nature and art may effect a cure without unusual interposition. To an insane person whose domestic attachments remain firm, and whose delusions in no way impair the moral affinity which should exist to those about him, removal to the society and care of strangers is a measure of, at best, questionable expediency."

One whose only offense may be is motly raiment, or, perhaps, the irregular way by which he supplies the wants nature has created within him, should not, even if he could, always be thus summarily dismissed from the sympathies of his fellows. And, even, if consigned to the hospital, he shortly reappears, from the necessity of the case, again to claim a seat at the Father's table. Society has yet a duty to learn toward the lunatic. His state has no kindred to crime, and should be regarded without aversion. He is not a fractional part as dangerous as he even appears. A few kind words, looks of confidence and sympathy, or a seat at the table, will smooth away the most dangerous features worn by any. It is mostly the neglect of society which has made him dangerous, and when this truth is fully learned he will be regarded with far different eyes than at present.

In a case not immediately urgent, discuss the idea fully among all these directly interested; examine well as to the value of the change to be made; and when a preponderance of opinion is secured, act decidedly and at once in using the agencies of the law. And when this decision is made, above all things we implore, that there be no deception used toward the one most to be affected by it. Inform him candidly and without disguise, that, by a common agreement among his best friends, he is to be placed for a while in the hospital, and, in the majority of instances, the expected opposition will not appear. It is better even that opposition be overcome by the judicious use of force, than by a fraud, however facile. There is, in the detestation of deception by the insane, something quite preternatural. It outlasts the remembrance of almost any other species of abuse. The complaint is almost daily heard here," "I can forgive them everything else except their deceiving me."

When these duties have been conscientiously performed, there is still another, not less important, best expressed in the sterling commandment, *Let him alone.* The difficulty in effecting a cure, in a patient, whose residence is in sight of the hospital windows, is often remarked by those having char ge of the insane. The human mind is the tenderest texture that passes through the Creative hand. Without the most subtle, precise, and guarded adjustment of treatment, its wounds are as difficult to heal as the torn filaments of gossamer.

We regret that our space will not permit further extracts from this valuable report.

---

*Eloquent Tribute to a Surgeon.*—In a sermon preached by Rev. Robert Collyer, of this city, in Nov. last, and which has been "published by some who heard it," we find the following language which we cannot forbear reproducing:

When I went to Fort Donelson to nurse our wounded men, it was my good fortune to be the personal attendant of a gentleman whose skill as a surgeon was only equalled by the wonderfully deep loving tenderness of his heart, as it thrilled in every tone of his voice and every touch of his hand. And it all comes up before me now, how he would come to the men, fearfully mangled as they were, and how the nerve would shrink and creep, and how with a wise, hard, steady skill he would cut to save life; forcing back tears of pity only that he might keep his eye clear for the delicate duty; speaking low words of cheer in tones heavy with tenderness; then when all was over, and the poor fellows, fainting with pain, knew that all was done that could be, and done with a severity whose touch was love, how they would look after the man as he went away, sending unspoken benedictions to attend him.

In a foot note the Reverend author observes:

My position as nurse for this gentleman, Dr. R. L. Rea, of this city, gave me such insight as inspired this poor tribute to his worth and goodness. He was one of a noble band, all full of the same spirit. I am glad to say such words of them, and all the more that I am sure they never expected to hear them.

Such mutual recognition by members of the different professions, elevate and inspire each to higher efforts in behalf of humanity.

We quote his language, all the more willingly, because so many of the "cloth" have disgraced themselves by studied attempts to degrade our profession and exalt all forms of quackery. It is unnecessary to say that rarely has compliment been better deserved than in this instance.

---

☞ At a meeting of the students of Rush Medical College, upon the decease of one of their number, the following resolutions were adopted:

WHEREAS, It hath pleased Him who watcheth the sparrow that not one fall to the ground without His care, to remove a respected and beloved fellow student, Chas. L. West, to the far distant spirit land "from whose bourne no traveler returns," therefore,

*Resolved*, That we, as a class, recognize in his decease the removal of one who gave promise of becoming a shining light in his chosen profession.

*Resolved*, That we tender to the brother and friends of the departed our heartfelt sympathy in this, their bereavement, and in this hour, when the cords with which nature has bound friend to friend are so untimely sundered.

*Resolved*, That a copy of the above be sent to the friends of the deceased and also to the Chicago *Medical Journal* for publication.

C. F. LITTLE,

G. ALLEN LAMB, } *Committee.*

F. EMMONS,

Chicago, Dec. 29th, 1862.

---

☞ Business letters to the JOURNAL should be addressed to Dr. E. INGALS, Chicago, Ills., P. O. Drawer 5787. When money is received a receipt will be returned with the next number of the JOURNAL, and subscribers failing to get such receipt will confer a favor by giving us notice of the omission.

---

VACCINE MATTER.—Fresh and reliable Vaccine Matter may be obtained by enclosing One Dollar and addressing

CHICAGO MEDICAL JOURNAL,
P. O. Drawer 5787, Chicago, Ill.

